# Vision-Based Structural Modal Identification Using Hybrid Motion Magnification

**DOI:** 10.3390/s22239287

**Published:** 2022-11-29

**Authors:** Dashan Zhang, Andong Zhu, Wenhui Hou, Lu Liu, Yuwei Wang

**Affiliations:** 1 College of Engineering, Anhui Agricultural University, Hefei 230036, China; 2 Anhui Province Engineering Laboratory of Intelligent Agricultural Machinery and Equipment, Anhui Agricultural University, Hefei 230036, China

**Keywords:** operational modal analysis, vision-based measurement, hybrid motion magnification, modal shapes visualization, temporal and spatial denoising

## Abstract

As a promising alternative to conventional contact sensors, vision-based technologies for a structural dynamic response measurement and health monitoring have attracted much attention from the research community. Among these technologies, Eulerian video magnification has a unique capability of analyzing modal responses and visualizing modal shapes. To reduce the noise interference and improve the quality and stability of the modal shape visualization, this study proposes a hybrid motion magnification framework that combines linear and phase-based motion processing. Based on the assumption that temporal variations can represent spatial motions, the linear motion processing extracts and manipulates the temporal intensity variations related to modal responses through matrix decomposition and underdetermined blind source separation (BSS) techniques. Meanwhile, the theory of Fourier transform profilometry (FTP) is utilized to reduce spatial high-frequency noise. As all spatial motions in a video are linearly controllable, the subsequent phase-based motion processing highlights the motions and visualizes the modal shapes with a higher quality. The proposed method is validated by two laboratory experiments and a field test on a large-scale truss bridge. The quantitative evaluation results with high-speed cameras demonstrate that the hybrid method performs better than the single-step phase-based motion magnification method in visualizing sound-induced subtle motions. In the field test, the vibration characteristics of the truss bridge when a train is driving across the bridge are studied with a commercial camera over 400 m away from the bridge. Moreover, four full-field modal shapes of the bridge are successfully observed.

## 1. Introduction

Structural experimental modal parameters, including modal frequencies, damping ratios, and modal shapes, provide insight into dynamic behaviors and are critical for applications such as structural health monitoring (SHM) and nondestructive testing (NDT) [1]. Usually, these properties are recovered by analyzing the vibrations of limited discrete points on the object through reliable contact sensors. However, the physically attached sensors may cause a mass-loading effect on lightweight targets, and they are difficult to affix to complex large-scale structures [2,3,4]. As an alternative, the vision-based method is one of the most popular non-contact measurement methods for the structural modal analysis in recent years [5,6,7]. Compared with common contact sensors, camera-based devices are more flexible and provide a higher spatial-resolution sensing capacity, which makes them convenient for remote installation and preferable for full-field measurements [8,9,10].

With advances in image processing techniques (e.g., image registration and optical flow), vision-based measurements can obtain intuitionistic image sequences of structural vibrations and are applied to experimental modal tests for various types of structures. In most cases [11,12], these methods extract the field deformation or local displacement from variations in speckle and high-contrast natural or artificial makers on the surface of the structure, which limits their applications to featureless and large-scale measurements. Meanwhile, although subpixel precision can be achieved, for extremely subtle motions, it is still difficult for algorithms to balance the efficiency and resolution, especially when full-field measurements are required.

As a computation technique for visualizing subtle colors and variations in videos, Eulerian video magnification [13,14,15] shows a strong vitality and is used in actual output-only modal analyses [16,17,18]. Unlike the motion extraction approaches based on an inter-frame correlation or gradient, the Eulerian approach considers that the structural in-plain small motion is closely related to the intensity or phase variations in the timeline. A quantitative analysis of these temporal variations reveals vital characteristics (e.g., the elasticity and modal frequency). Moreover, by manipulating the spatial motion, structural modal shapes can be directly observed in motion-magnified videos [19,20,21,22,23,24].

Although magnifying the temporal intensity or phase variations can achieve motion magnification, these two frameworks have different characteristics. Normally, linear processing is more sensitive to subtle motions and less robust to the noise from imaging sensors and illumination. Meanwhile, phase-based motion processing performs better in noise control and can support a larger amplification factor, so it is more suitable for visualizing and understanding modal shapes. However, both spatial and temporal noises severely affect the quality of the outputs of phase-based motion processing, especially in a subtle and long-distance motion observation [18,19,20,24,25]. In practice, it is difficult to uniformly reduce the temporal phase noise without any prior information of the measured structures. In addition, the existence of multi-scale decomposition also increases the complexity of the noise processing in both space and timeline [14,18,23].

To reduce noises and improve the quality of the modal shape visualization, it is desirable to propose a hybrid motion magnification framework that combines linear and phase-based motion processing. Based on the assumption that temporal variations can approximate spatial motions, previous studies [26] have shown that the singular value decomposition (SVD) can extract the structural vibration from the temporal intensity variations, and the spatial motions in videos can be manipulated linearly with a higher efficiency. Considering that the extracted temporal variations relevant to vibrations are mixed signals, the sparse component analysis (SCA) technique is used in signal separation [27,28]. Meanwhile, as noises mainly exist in the high-spatial-frequency part [13,14,15], Fourier transform profilometry (FTP) is utilized to improve the weights that represent the severity of the spatial motion [29,30]. In the hybrid framework, linear motion processing simplifies the processes of vibration extraction and noise reduction and provides high-quality, controllable inputs for visualizing modal shapes in phase-based motion processing. The proposed framework was applied to two laboratory experiments and a field test on a large-scale truss bridge to evaluate its performance in a modal analysis.

The main contributions of this paper are summarized as follows: (1) A linear motion processing approach is proposed to extract and manipulate the structural vibrations in videos. Meanwhile, a set of methods is developed to reduce the temporal and spatial noises. (2) The high-quality visualization of structural modal shapes is realized in the hybrid motion magnification framework. (3) The performance of the proposed framework is investigated through sound-induced modal tests in the laboratory. The effectiveness of this proposed framework is verified in a long-distance field test to analyze the vibration characteristics of a large-scale truss bridge.

The rest of this paper is organized as follows. Section 2 introduces the proposed hybrid motion magnification framework, including the details of the temporal and spatial noise reduction. The experimental data with a lightweight beam from the MIT CSAIL [19] are analyzed to better understand the implementation scheme. Section 3 validates the proposed method through a set of experiments and discusses its advantages and limitations. Section 4 concludes this paper.

## 2. Materials and Methods

### 2.1. Structural Vibration and Intensity Variations

Modal analysis models a solid object as a system of point masses connected by springs and dampers. Without loss of generality, the differential equation of a multi-DOF vibration system is expressed as

(1) $$M\ddot{p}+C\dot{p}+Kp=0,$$

where M is the mass matrix; C and K are matrices describing the viscous damping values and spring stiffness between points, respectively; *p*, $\dot{p}$, and $\ddot{p}$ are vectors indicating the displacement, velocity, and acceleration of the points, respectively. Under the assumption of Rayleigh damping, matrix C is ideal and is assumed to be a linear combination of M and K. After the generalized eigenvalue problem is solved, the system can be decoupled into single-degree freedom systems, and the vibration motion of modal masses can be expressed as a linear combination of the modal responses:

(2) $$p\left(t\right)=\Phi q\left(t\right)=\sum_{i=1}^{n}\phi_{i}q_{i}\left(t\right),$$

where *n* is the mode number; Φ is the modal matrix that defines modal coordinates $q\left(t\right)$; $\phi_{i}$ and $q_{i}$ are, respectively, the *i*-th mode shape and modal coordinate.

With the assistance of imaging, structural vibration can be measured by video records containing frames with temporally translated image intensities. From the Eulerian perspective, temporal filtering can approximate spatial translation [13,14,15,19]. To investigate the relationship between intensity variations and vibration, the simple case of 1D signal translation is considered in this paper. Let I(x,t) be the image intensity at position *x* and time *t*. The observed intensity variations can be expressed by a displacement function δ(x,t), and δ(x,0)=0. All valuable intensity variations $\tilde{\delta }\left(x,t\right)$ should be highly correlated with the modal responses:

(3) $$\tilde{\delta }\left(x,t\right):=\sum_{i=1}^{n}w_{i}\left(x\right)q_{i}\left(t\right),$$

where $w_{i}\left(x\right)$ is the weight corresponding to the *i*-th mode shape (related to modal coordinates). Thus, the displacement function δ(x,t) is expressed as the combination of $\tilde{\delta }\left(x,t\right)$ and noise:

(4) $$\delta \left(x,t\right)=\sum_{i=1}^{n}w_{i}\left(x\right)q_{i}\left(t\right)+N\left(x,t\right),$$

where N(x,t) is the noise mainly caused by the environment and imaging.

### 2.2. Linear Motion Processing

From Equation (4), to achieve linear motion magnification at a particular resonant frequency, $\tilde{\delta }\left(x,t\right)$ needs to be estimated and decoupled, and the noise N(x,t) needs to be reduced at every pixel coordinate:

(5) $${\tilde{I}}_{i}\left(x,t\right)=I\left(x,t\right)+\alpha_{i}\left(w_{i}\left(x\right)q_{i}\left(t\right)+n_{i}\left(x,t\right)\right),$$

where $\alpha_{i}$ is the amplification factor for the *i*-th mode, and $n_{i}\left(x,t\right)$ is the residual noise ($n_{i}\left(x,t\right)\ll N\left(x,t\right)$).

**Figure 1:**
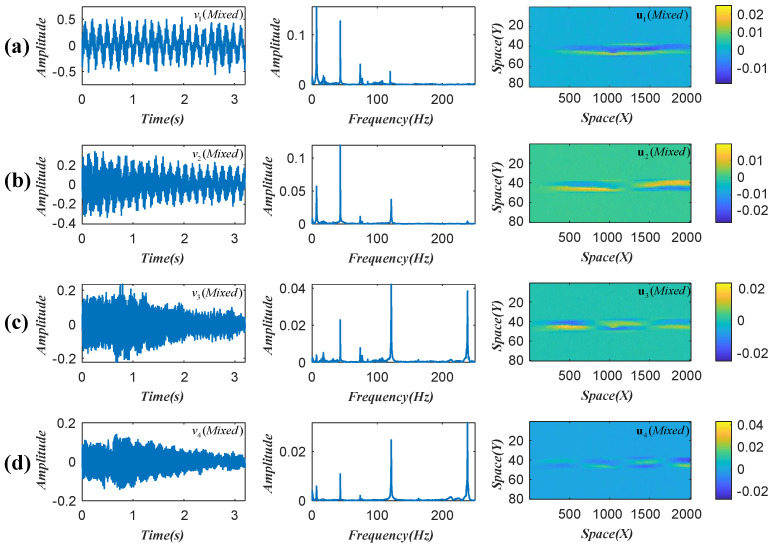
Reserved temporal intensity variations and corresponding weights in beam test (**a**–**d**).

Based on the assumption that useful intensity variations and noises are linearly independent, $\tilde{\delta }\left(x,t\right)$ and N(x,t) can be separated by using SVD efficiently [26]. For each pixel coordinate, the difference between I(x,t) and I(x,0) is calculated and then used to reshape matrix D that represents the temporal intensity variations in the video. Through SVD, this matrix is decomposed, and *k* significant singular values are reserved:

(6) $$D_{c\times l}\underset{SVD}{\to }U_{c\times k}\cdot S_{k\times k}\cdot V_{l\times k}^{*}=\sum_{r=1}^{k}u_{r}s_{r}v_{r}^{*},$$

where *c* is the total number of pixel coordinates; *l* is the length of the video; $s_{r}$ is reserved singular value; $u_{r}$ and $v_{r}$ are, respectively, orthogonal left-singular and right-singular vectors; symbol * means matrix transposition. The reserved $s_{r}v_{r}^{*}\left(r=1,2,...,k\right)$ are considered as the output observations representing an instantaneous linear mixture of signals $q_{i}\left(t\right)\left(i=1,2,...,n\right)$:

(7) $$\left[\begin{array}{c} s_{1}v_{1}^{*}\\ s_{2}v_{2}^{*}\\ \vdots \\ s_{k}v_{k}^{*} \end{array}\right]=A\left[\begin{array}{c} q_{1}\left(t\right)\\ q_{2}\left(t\right)\\ \vdots \\ q_{n}\left(t\right) \end{array}\right],$$

where A is referred to as the mixing matrix. The reserved four temporal intensity variations, their frequency spectra, and the corresponding weights are illustrated in Figure 1. It can be seen that the reserved intensity variations are coupled signals of multiple modal responses [19].

Taking Equation (7) as an operational modal analysis (OMA) problem, the mixing matrix can be estimated by using the blind source separation (BSS) technique. The well-posedness of Equation (7) is determined by the magnitude of *k* (the number of reserved singular values) and *n* (the activated maximum mode order). In this paper, the equation is considered an underdetermined BSS problem, and mixing matrix A is estimated via SCA [27,28]. By decoupling the reserved intensity variations, the corresponding weights are updated as follows:

(8) $${\tilde{u}}_{i}=\sum_{r=1}^{k}u_{r}s_{r}v_{r}^{*}\times q_{i}{\left(t\right)}^{*}.$$

Thus, according to Equation (5), the output of linear motion processing can be expressed as

(9) $$\begin{array}{c} {\tilde{I}}_{i}\left(x,t\right)=I\left(x,t\right)+\alpha_{i}\left({\tilde{u}}_{i}\left(x\right)q_{i}\left(t\right)\right)\approx I\left(x,0\right)+\sum_{i=1}^{n}w_{i}\left(x\right)q_{i}\left(t\right)+N\left(x,t\right)+\alpha_{i}\left({\tilde{u}}_{i}\left(x\right)q_{i}\left(t\right)\right). \end{array}$$

To further reduce the noises and remove the existing vibrations in the input video, this process can be performed on the first frame of the video sequence, i.e.,

(10) $${\tilde{I}}_{i}\left(x,t\right)=I\left(x,0\right)+\alpha_{i}\left({\tilde{u}}_{i}\left(x\right)q_{i}\left(t\right)\right).$$

**Figure 2:**
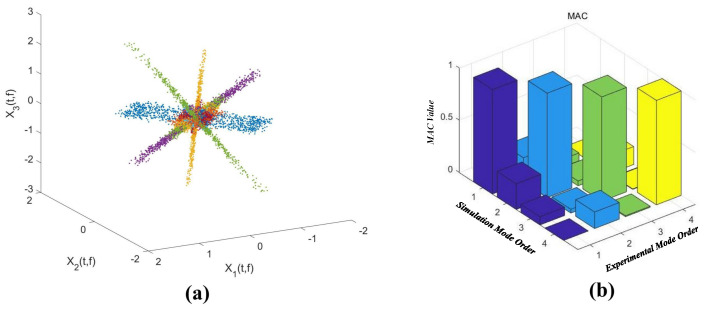
(**a**) Scatter diagram of the first three measuring signals and (**b**) the modal assurance criterion (MAC).

**Figure 3:**
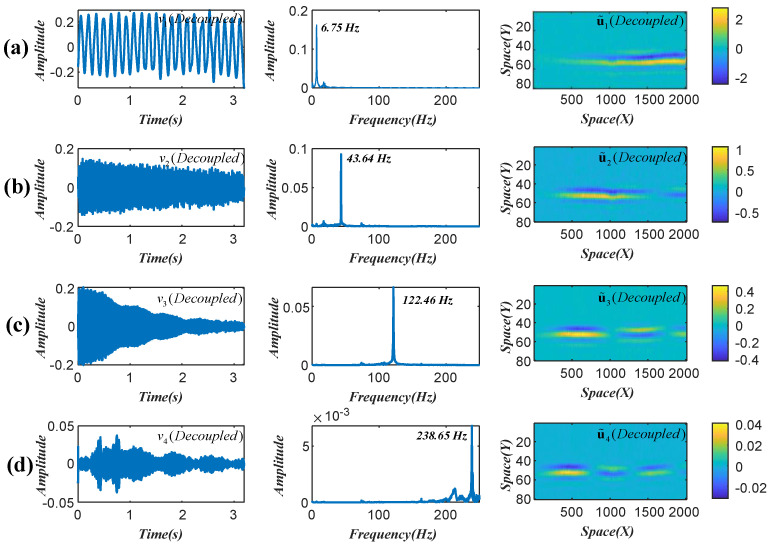
Decoupled temporal intensity variations and corresponding weights in beam test. (**a**) First mode; (**b**) second mode; (**c**) third mode; and (**d**) fourth mode.

Figure 2a shows the scatter diagram of the first three temporal intensity variations ($s_{r}v_{r}^{*}\left(r=1,2,3\right)$). The modal assurance criterion (MAC) in Figure 2b is used to determine the errors of the estimated mode shape vectors. The observed directions in Figure 2a represent the estimated four mode shape vectors, and the theoretical mode shape vectors are calculated by using the FEM software. The time-domain modal responses are recovered by using the $l_{1}$-optimization algorithm [27]. The decoupled temporal intensity variations, their frequency spectra, and the updated weights are illustrated in Figure 3. It is considered that these decoupled temporal intensity variations are highly correlated with the first four modal responses [19].

### 2.3. Weight Enhancement of the FTP

According to Equation (8), the spatial weights ${\tilde{u}}_{i}$ are calculated by using the decoupled intensity variations. As the linear motion processing above does not consider the spatial consistency, the updated weights ${\tilde{u}}_{i}$ are not spatially smooth and continuous. As noises mainly exist in high spatial-frequency temporal variations, the FTP is utilized to improve the quality of spatial weights [29,30].

**Figure 4:**
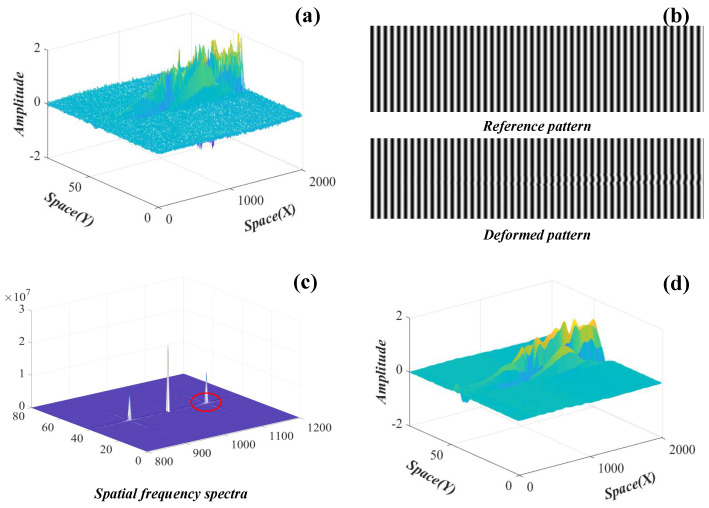
The spatial weight enhancement process of ${\tilde{u}}_{1}$. (**a**) The spatial weight ${\tilde{u}}_{1}$; (**b**) the reference and deformed grating images; (**c**) the spatial-frequency spectra of the deformed grating image; (**d**) the improved spatial weight ${\hat{u}}_{1}$.

Taking ${\tilde{u}}_{1}$ as an example, Figure 4 shows the enhancement process. Let the spatial weights ${\tilde{u}}_{1}$ (Figure 4a) deform the reference grating image, the deformed grating image is shown in Figure 4b, and the spatial-frequency spectra of the deformed grating image are shown in Figure 4c. Assuming that the noise is mainly related to the high-frequency component in the spatial-frequency spectra, the spectra are filtered to let only the fundamental component through (red circle), and then reversed Fourier transform is applied to the fundamental component. According to the theory of FTP, the core variable that varies directly with the spatial weights is the phase distribution. The formula to obtain the improved spatial weights is given as

(11) $${\hat{u}}_{i}=\frac{l_{0}\Delta \eta_{i}}{\Delta \eta_{i}-2\pi f_{0}d},$$

where $\Delta \eta_{i}$ is the unwrapped phase difference; $f_{0}$ is the fundamental frequency of the observed grating image; $l_{0}$ and *d* are preset values in the crossed-optical-axes geometry of FTP. The improved spatial weights ${\hat{u}}_{1}$ are shown in Figure 4d.

**Figure 5:**
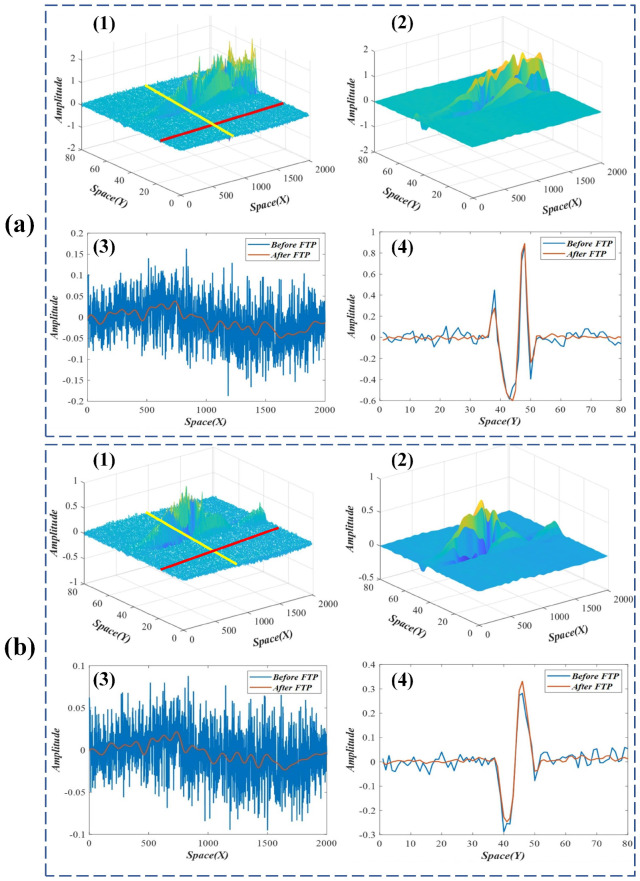
(**a**,**b**), the FTP results and analyses of the first two modes in the beam test. (1) Original spatial weights; (2) improved spatial weights; (3) and (4), sampling results of the original and the improved weights in *x* and *y* directions.

**Figure 6:**
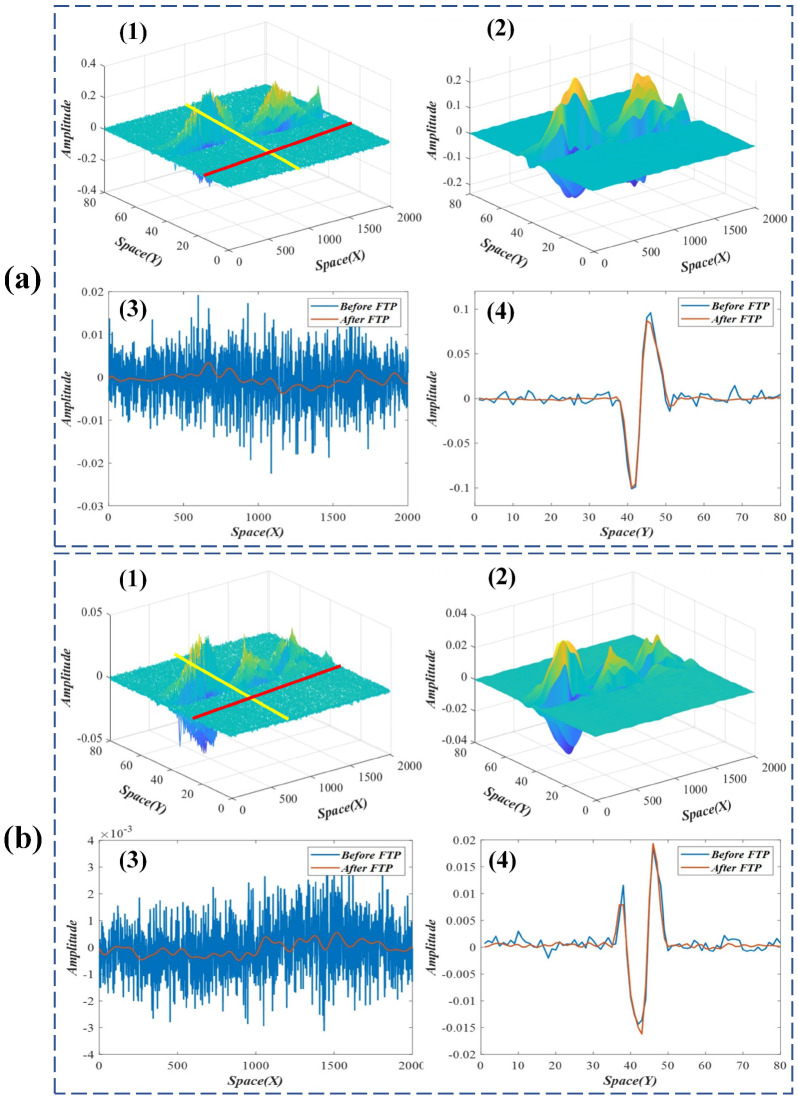
(**a**,**b**), the FTP results and analyses of the third and fourth modes in the beam test. (1) Original spatial weights; (2) improved spatial weights; (3) and (4), sampling results of the original and the improved weights in *x* and *y* directions.

The results and analyses of the improved spatial weights in the beam test are presented in Figure 5 and Figure 6. Subfigures (1) and (2) in Figure 5 and Figure 6 compare the original and the improved spatial weights. It can be seen that the spatial weights improved by FTP are much smoother than the originals and have better performance on spatial consistency. Subfigures (3) and (4) in Figure 5 and Figure 6 compare the sampling results of the original and the improved weights in different spatial directions (the red and yellow lines in subfigure (1)). The results indicate that the noises in improved spatial weights are significantly reduced.

**Figure 7:**
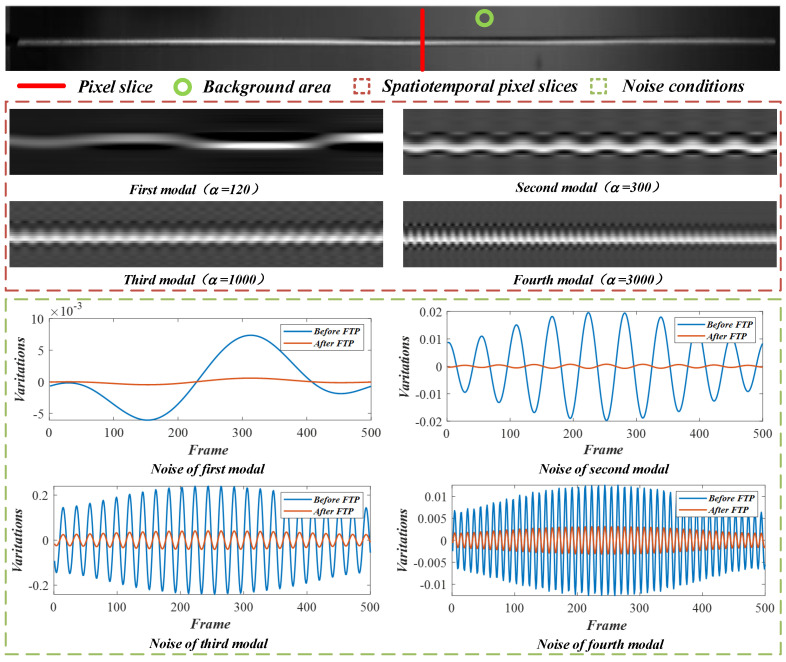
Linear motion processing results and noise reduction analysis in the beam test.

According to Equation (10), linear motion magnification can be achieved by using the decoupled temporal intensity variations and improved spatial weights. Figure 7 illustrates the linear motion magnification results in the beam test of MIT CSAIL [19]. The effectiveness of linear motion processing is validated with the spatiotemporal pixel slices cut from the motion-magnified videos. The mean intensity values inside the green circle in the background are calculated to study the residual noise. The analysis results indicate that these motion-magnified videos obtained by using the improved spatial weights achieve better performance on noise control.

### 2.4. Phase-Based Motion Processing

The reason for further phase-based motion processing is that this framework can support large amplification factors and show better noise performance than the linear approximation. From Equation (10), the structural vibrations in the video can be initially produced through linear motion processing. Based on phase-based motion processing, the produced image profile can be decomposed into a sum of complex sinusoids by using the Fourier series:

(12) $${\tilde{I}}_{i}\left(x,t\right)=\sum_{\omega =-\infty }^{\infty }B_{\omega }e^{j\omega \left(x+\alpha_{i}{\hat{u}}_{i}\left(x\right)q_{i}\left(t\right)\right)}.$$

Let ${\tilde{\delta }}_{i}\left(x,t\right)={\hat{u}}_{i}\left(x\right)q_{i}\left(t\right)$ denote the initial motion. The band corresponding to a single frequency ω is the complex sinusoid:

(13) $$S_{\omega }\left(x,t\right)=B_{\omega }e^{j\omega \left(x+\alpha_{i}{\tilde{\delta }}_{i}\left(x,t\right)\right)}.$$

Because the initial motions in the video are controlled according to specific mode shapes, in phase-based motion processing, only the temporal DC component [14] of the phase $\omega \left(x+\alpha_{i}{\tilde{\delta }}_{i}\left(x,t\right)\right)$ needs to be removed. Then, the phase $\alpha_{i}{\tilde{\delta }}_{i}\left(x,t\right)$ is multiplied with another amplification factor $\beta_{i}$ to obtain the motion magnified sub-band:

(14) $$\begin{array}{c} {\tilde{S}}_{\omega }\left(x,t\right)=S_{\omega }\left(x,t\right)e^{j\alpha_{i}\beta_{i}\omega {\tilde{\delta }}_{i}\left(x,t\right)}=B_{\omega }e^{j\omega \left(x+\left(1+\beta_{i}\right)\alpha_{i}{\tilde{\delta }}_{i}\left(x,t\right)\right)}. \end{array}$$

The motion-magnified sequence can be eventually reconstructed by summing all the sub-bands. The total magnification factor in Equation (14) is $\left(1+\beta_{i}\right)\alpha_{i}$.

**Figure 8:**
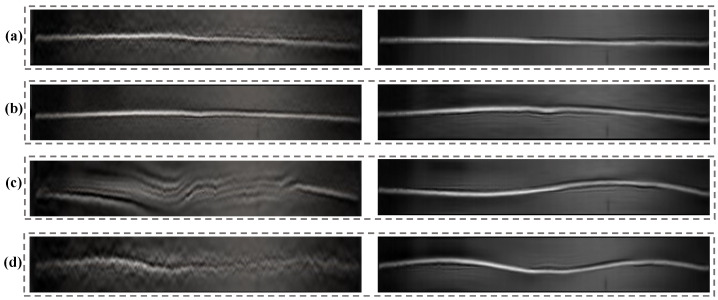
The comparison between the original phase-based method and our improved method. (**a**) The first mode; (**b**) the second mode; (**c**) the third mode; and (**d**) the fourth mode. More results are shown in the Supplementary videos.

The motion magnification results of the original phase-based method and our improved framework are compared, and the result is shown in Figure 8 (8 orientations, half-octave bandwidth pyramids). The filter bands of the original phase-based approach are set to ±2 Hz near the experimental modal frequencies of the test beam. Table 1 presents the magnification factors and compares the image quality results of the reconstructed videos. The results of the average blind/referenceless image spatial quality evaluator (BRISQUE) [31] indicate that these videos reconstructed by the improved framework have better image quality. The average BRISQUE score of the input video image is 41.98. Because the initial motion is 0 (Equation (10)), the overall amplification factors of our improved framework are larger than those used in the original.

## 3. Results

### 3.1. Vibration Analysis of a Lightweight Beam

**Figure 9:**
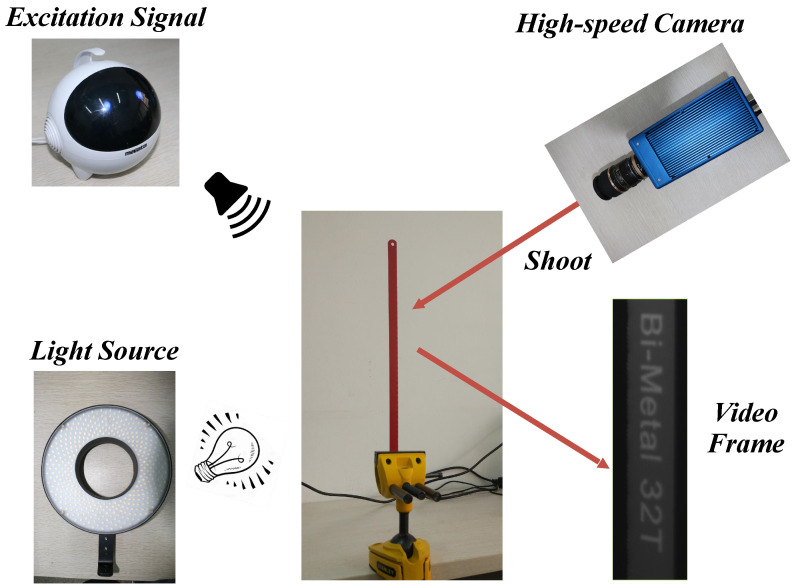
Experiment setup of the laboratory lightweight beam test.

In the first case, the modal parameters of a lightweight beam from a video are analyzed in a controlled laboratory experiment. The experiment setup is illustrated in Figure 9. The lightweight beam made of alloy steel was clamped with a table vice. During the experiment, audio with a frequency band ranging from 10 to 500 Hz was played by the loudspeaker about 0.1 m away from the surface of the beam at 80 decibels. When air fluctuations reach the beam, subtle forced vibrations will appear on the surface. Meanwhile, subtle vibrations motivated by the excitation audio were recorded by the high-speed camera system (Revealer 5KF10M, Agile Device Inc., Hefei, China) at 500 fps with a resolution of 580 × 180 pixels. The dimension and the material parameters of the beam are listed in Table 2. According to the Euler–Bernoulli beam theory, the theoretical resonant frequencies of a cantilever beam are estimated as follows:

(15) $$f_{n}=\frac{3.52\gamma }{2\pi l^{2}}\sqrt{\frac{ER}{\rho A}},\left(\gamma =1,6.27,17.55,34.39...\right),$$

where $f_{n}$, *E*, and *R* denote the resonant frequency of the *n*-th mode, Young’s Modulus, and the moment of inertia of the beam, respectively; ρ, *A*, and *l* denote the density, the cross-sectional area, and the length of the beam, respectively.

**Figure 10:**
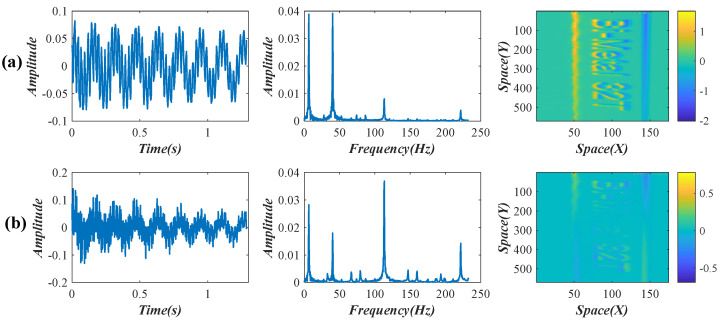
(**a**,**b**), reserved temporal intensity variations, their frequency spectra, and the corresponding weights in the lightweight beam test.

**Figure 11:**
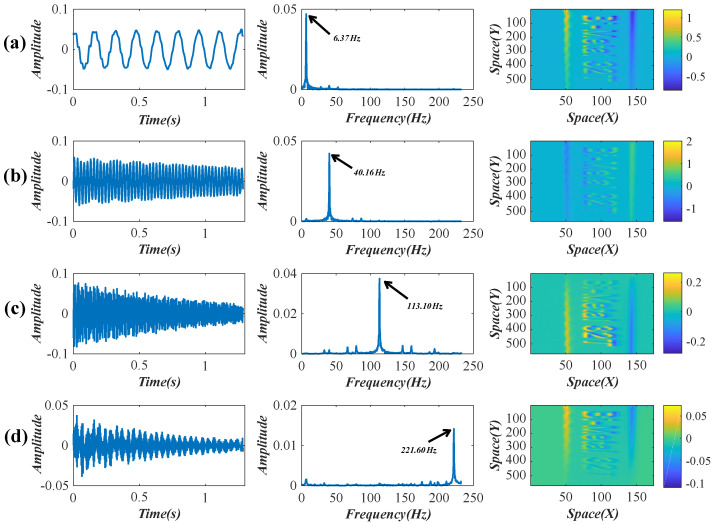
(**a**–**d**) Decoupled temporal intensity variations, their frequency spectra, and the corresponding weights (enhanced by FTP) in the lightweight beam test.

After the SVD decomposition, two temporal intensity variations were reserved. Their waveforms, frequency spectra, and the corresponding weights are shown in Figure 10. By decoupling the two reserved intensity variations through an SCA, four obvious peaks, including 6.37, 40.16, 113.10, and 221.60 Hz, were detected from the power spectra of the decoupled signals. According to Equation (15), these four temporal intensity variations are connected with the subtle spatial motions of the first four modal shapes. The comparison between the theoretical and experimental resonant frequencies is illustrated in Table 3. The decoupled intensity variations, their frequency spectra, and the updated weights (enhanced by FTP) are shown in Figure 11.

**Figure 12:**
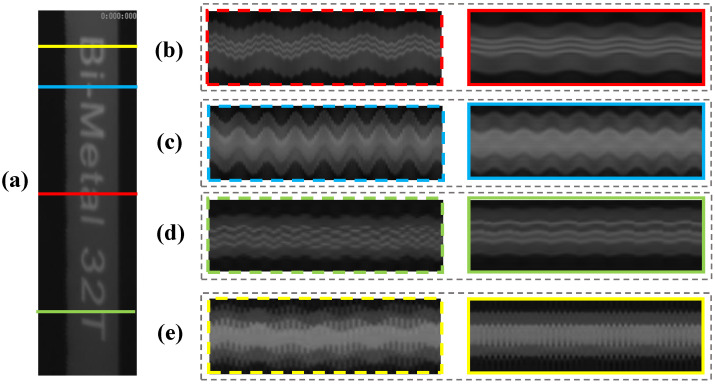
(**a**) Video frame and location of spatiotemporal slices and (**b**–**e**) spatiotemporal slices comparisons between the original and our improved framework in the lightweight beam test. More results are shown in the Supplementary videos.

After the decoupled temporal intensity variations and the corresponding spatial weights were obtained, the motions in the video frames can be produced linearly and then magnified through the phase-based processing. The complex steerable pyramids (eight orientations, half-octave bandwidth pyramids) were used to decompose the video frames, and the local phases in different spatial scales and orientations over time were obtained. The filter bands for the original phase-based magnification were set to ±2 Hz near the experimental modal frequencies. Figure 12 compares the final motion-magnified videos obtained by the original and our improved framework. The four colored lines in Figure 12a indicate the locations of the spatiotemporal pixel slices. Figure 12b–e) show the spatiotemporal slices of the first to the fourth modal shapes, respectively. It can be seen that the beam in the video reconstructed by our framework (solid line boxes) vibrates properly following a specific vibration mode, and the existing motions in the input video are removed. Table 4 presents the magnification factors and compares the image quality of the processed videos. The average BRISQUE score of the input video image is 39.34. The motion-magnified videos of the improved framework achieve a better image quality.

### 3.2. Vibration Analysis of the Nanfeihe Truss Bridge

The modal parameters of bridges reflect their vibration characteristics and are significant for bridge design and a structural state assessment. For large-scale bridges, it is difficult to excite the heavy structure with traditional vibration excitation devices and obtain structural vibration modes. In the second experiment, the vibration of the Nanfeihe railway truss bridge is observed under the wind–train–bridge coupling condition with a commercial camera, and the four modal shapes of the bridge are visualized with our improved motion magnification framework.

**Figure 13:**
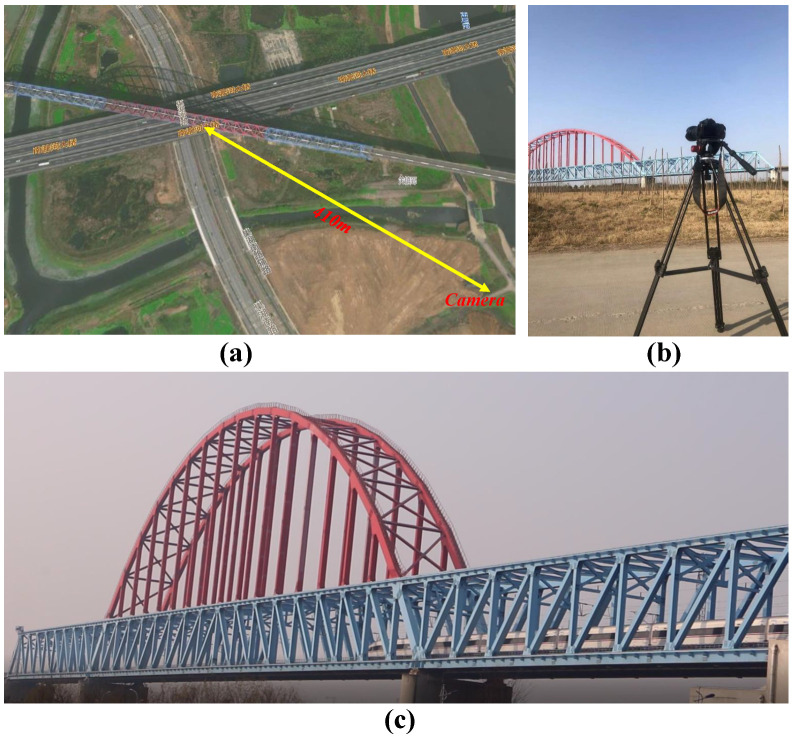
(**a**) The distance between camera and center of the bridge (image ©Baidu), (**b**) camera system, and (**c**) selected ROI.

The Nanfeihe railway truss bridge is a super-spanned railway bridge about 8 km away from Hefei South Railway Station. The bridge is a low-supported steel bridge composed of a continuous truss and flexible arch with a main span of 229.5 m. The rise of the arch is 45 m, and the overall length of the bridge is 461 m. The weight of the bridge is over 13,000 tons. As shown in Figure 13, a commercial camera (Canon 70D) was installed about 410 m away from the center of the bridge by the Nanfei river. Figure 13a illustrates the satellite view of the camera measurement location relative to the bridge. The camera and the bridge in the same view are shown in Figure 13b. During the filming, a high-speed train was driving across the bridge. The camera recorded the whole process at 25 fps with the resolution of 1920 × 1080 pixels. A 645 × 1775 pixel region of interest (ROI) was selected from the screenshot to reduce the interference of the background (Figure 13c).

For the data analysis, it is necessary to discuss and specify the vibration situation of the truss bridge. Before the high-speed train arrives, the vibration of the bridge is mainly caused by the environmental wind load. When the train arrives on the bridge, deflection appears on the structure, and the bridge will be affected by the excitation of both the wind and the train. For simplicity, this paper assumes that the two vibration processes (forced by the wind and the wind and train) are steady, compelled vibration processes and then discusses the influence of the transient vibration.

**Figure 14:**
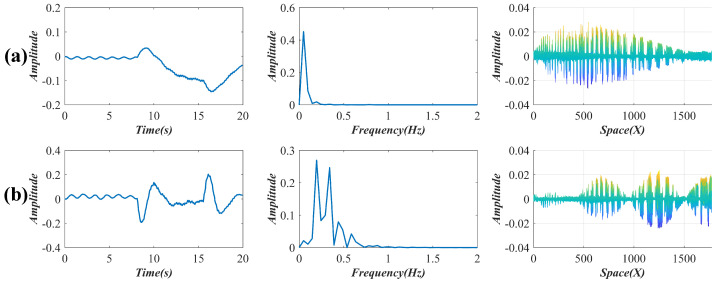
(**a**,**b**), reserved temporal intensity variations, their frequency spectra, and the corresponding weights in the bridge test.

**Figure 15:**
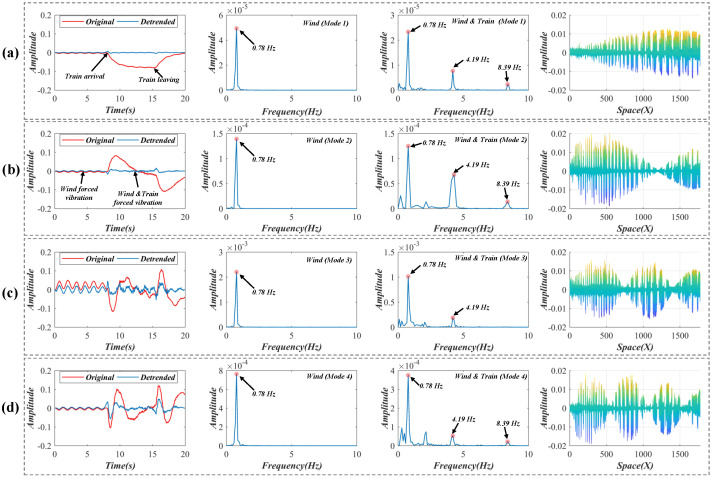
(**a**–**d**) Decoupled temporal intensity variations, power spectra under different excitations and weights in the bridge test. The first column represents the original and detrended reserved variations. The second and third columns show the power spectra before and train arrival time. The last column shows the decoupled weights in the bridge test.

Figure 14 shows the two reserved temporal intensity variations after the SVD decomposition of the pixel difference matrix from the video file. As shown in the first column in Figure 15, four independent components (red curves) are decoupled from the reserved variations through an SCA. When the train arrives on the bridge, large variations that reflect the bridge deflections appear on the curves. The train arrival time and train leaving time are marked by arrows in Figure 15a and are found at corresponding positions in Figure 15b–d. To investigate the influence of deflections, this paper detrends these intensity variations (blue curves) and then separates the signals according to the difference in the excitation source before a frequency analysis. The second and the third columns in Figure 15 show the power spectra of the detrended intensity variations before and after the train arrives. The vibration frequency under the load of the wind is 0.78 Hz, and the main frequency under the excitation of the train is 4.19 and 8.39 Hz (a multiple frequency of 4.19 Hz) [32,33,34]. The updated spatial weights are illustrated in the last column of Figure 15. From the angle perpendicular to the image plane, these spatial weights exhibit the four different vibration modes of the bridge. It is worth mentioning the FTP was not utilized here because the frequency distribution of the spatial weights was too complex to be separated by the FTP.

**Figure 16:**
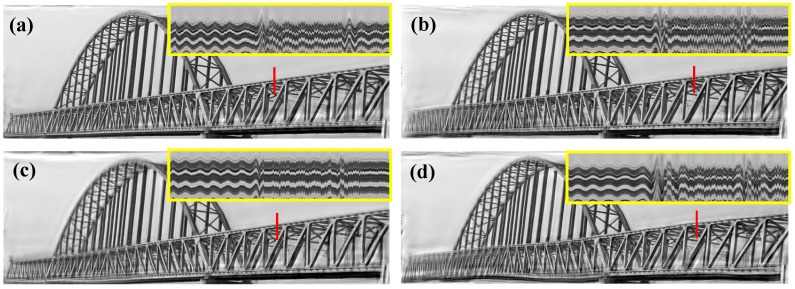
(**a**–**d**) Motion magnification results in the truss bridge test (8 orientations, quarter-octave bandwidth pyramids). More results are shown in the Supplementary videos.

After the linear and phase-based motion processing, four videos that reflect the different modal shapes were reconstructed. The amplification factors in the linear and phase-based motion processing are illustrated in Table 5, and the screenshots of the four motion-magnified videos are shown in Figure 16. The magnification factors are restricted to avoid too many artifacts or blurs, and the motion of the different modal shapes can be better perceived from the video files. Because the motion corresponding to a specific mode cannot be separated simply by temporal filtering, the results of the original phase-based method are not presented here for predictable modal aliasing.

**Figure 17:**
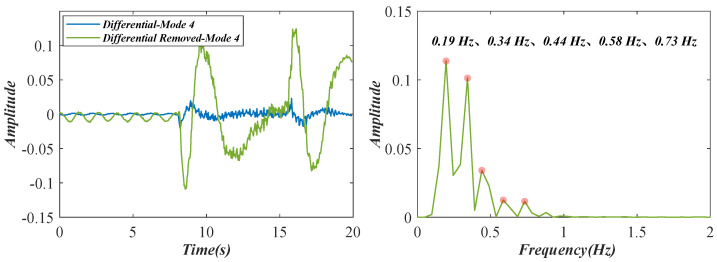
Damped vibration analysis result of the Nanfeihe truss bridge (the fourth variations).

When the train was driving across the bridge, a large deflection appeared on the bridge and then was attenuated by structural damping. For a simple single-DOF system, the transient vibration process is expressed as the combination of the damped vibration and the equal-amplitude vibration [35,36]. Due to the variation in the load, the attenuation of the deflection is in an unsteady state. Therefore, the differential of the decoupled temporal intensity variations is removed from the original data to investigate the influence of the damped vibration on the system. Figure 17 shows the results of the detected fourth variations (in Figure 15d). Several low-frequency peaks at 0.19, 0.34, 0.44, 0.58, and 0.73 Hz are found in the power spectrum. These peaks may be the resonant frequencies of the test bridge.

## 4. Discussion

According to the theory of Eulerian video magnification, the spatial motion of a structure can be linearly approximated as temporal pixel variations. In hybrid motion processing, linear motion processing provides an effective approach to separate valuable temporal pixel variations and their corresponding spatial weights through an SVD and SCA. The FTP is utilized to improve the spatial weight matrices to achieve a better spatial consistency and noise reduction effect. Although the presented framework performs better on the vibration analysis than Eulerian linear processing, these two approaches have common limitations of a relatively low amplification factor and noise amplification. Therefore, the output of linear motion processing is usually taken as the controllable input of the following phase-based motion processing. In practical applications, to minimize the residual noise, the motions generated in the pixel domain would be better to be just recognized enough by temporal phase variations, indicating that the factor $\alpha_{i}$ should not be too large. As spatial motion is generated into video, all temporal phase variations are usable, so temporal filtering can be omitted in phase-based motion processing. It is worth noting that for certain spatial motions, the phase amplification factor is still restricted by the spatial wavelength and the number of filters per octave for each orientation [14,15]. Therefore, the overall amplification factor in phase-based motion processing is not extended. As high-frequency components in images cannot be pushed as far as low-frequency components, breaking the restriction of the phase amplification factor will lead to artifacts or blurs. In the truss bridge test, blurs and artifacts are allowed to achieve a better perception of the modal shape.

Considering the fact that noises mainly exist in the high spatial-frequency part of the spatial weight, the FTP reserves a globally low spatial frequency of the spatial weights rather than directly reducing the amplification of these high spatial-frequency temporal variations [13,15]. In the current linear motion processing, the noise reduction process is simple and efficient in these controlled laboratory experiments without involving multi-scale decomposition. However, in the practical long-distance bridge test, the image quality is severely reduced due to the changing lighting and background conditions (e.g., clouds and the appearance of the high-speed train) [25]. This makes the FTP inefficient. The problem may be alleviated by setting masks for all video frames, but the whole process is too laborious, especially for high-speed videos. Moreover, in the field test, it is critical to remove the existing apparent motions in the video (Equation (10)) to ensure the stability of the modal shape visualization [20,25]. Based on the above analysis, we will attempt to address these issues in our future work and explore the practicability of the proposed framework for complex engineering structures.

## 5. Conclusions

In this paper, a hybrid motion processing framework that combines linear and phase-based motion processing is proposed, and its performance is evaluated through structural modal tests. By extracting, denoising, and manipulating the temporal intensity variations that are closely related to modal responses, the linear motion processing provides controllable, high-quality input for the subsequent phase-based motion processing, thus greatly improving the presentation of modal shapes. The proposed method is verified by two laboratory experiments on lightweight beams and a field test on a truss bridge. The experimental results indicate that the proposed motion processing framework can alleviate noise interference and obtain good results in subtle and long-distance motion observation. It should be pointed out that in the measurement of complex structures with a single camera, the motions in the image plane are considered as the projection of 3D vibration. Accurately representing global 3D motions of complex, large-scale engineering structures is challenging and significant. In addition to the issues listed in Discussions, we will further study the visualization of modal shapes in 3D space by extending the concept of motion amplification to 3D dynamic measurement techniques, such as multi-camera and structured light systems.

## Figures and Tables

**Table 1:** The amplification factors and image quality in the beam test.

Mode	Factor	Factor	Factor	BRISQUE	BRISQUE
Order	(Original)	($\alpha_{i}$)	($\beta_{i}$)	(Original)	(Improved)
1st	350	10	40	50.94	45.48
2nd	600	30	25	46.78	44.13
3rd	1000	100	15	50.20	44.30
4th	12,000	1000	20	56.00	43.69

**Table 2:** Parameters of the lightweight beam.

Dimensions (mm)	Young’s Modulus	Density
290×12.6×0.65	$2.06\times 10^{11}$ N·m^−2^	$7.85\times 10^{3}$ kg·m^−3^

**Table 3:** Comparison between the theoretical and experimental resonant frequencies in the lightweight beam test.

Mode Order	Theoretical (Hz)	Experimental (Hz)	Error Rate (%)
1st	6.41	6.37	0.62
2nd	40.17	40.16	0.02
3rd	112.43	113.10	0.59
4th	220.31	221.60	0.08

**Table 4:** The amplification factors and image quality in the case of the lightweight beam.

Mode	Factor	Factor	Factor	BRISQUE	BRISQUE
Order	(Original)	($\alpha_{i}$)	($\beta_{i}$)	(Original)	(Improved)
1st	10	0.15	100	50.84	40.45
2nd	15	0.2	200	52.10	41.58
3rd	60	2	50	48.04	41.75
4th	100	5	30	49.24	40.78

**Table 5:** Amplification factors in the Nanfeihe truss bridge case.

**Mode Number**	1	2	3	4
**Linear ($\alpha_{i}$)**	10	15	25	30
**Phase-based ($\beta_{i}$)**	400	400	800	800

## Data Availability

Data sharing is not applicable to this article.

## Supplementary Materials

The following are available online at https://www.mdpi.com/article/10.3390/s22239287/s1, Supplementary videos for Figure 8, Figure 12 and Figure 16.
